# Coronary vasospasms induced by a dexmedetomidine infusion for deep sedation during catheter ablation in a patient with negative findings of an acetylcholine provocation test

**DOI:** 10.1002/joa3.12918

**Published:** 2023-08-24

**Authors:** Wataru Sasaki, Kenta Tsutsui, Takahide Arai, Hitoshi Mori, Ritsushi Kato

**Affiliations:** ^1^ Department of Cardiology Saitama Medical University International Medical Center Hidaka Japan

**Keywords:** catheter ablation, coronary vasospasm, dexmedetomidine

Catheter ablation (CA) is an effective treatment for atrial fibrillation (AF) and has been widely performed. Coronary vasospasms have been reported as a rare complication during CA in which coronary angiography (CAG) shows severe stenosis that is dilated to normal by a coronary injection of nitroglycerin.^1^, ^2^, ^3^ A multicenter study in Japan demonstrated that the incidence of coronary vasospasms during CA was 0.19%.^1^ A common cause is energy deliveries during RF applications. For example, the cavo‐tricuspid isthmus (CTI) is close to the right coronary artery (RCA), and ablation of the CTI induces RCA spasms.^4^ However, on rare occasions, coronary vasospasms occur before beginning the RF application, suggesting a potential role of sedatives in the development of coronary vasospasms. The previous research that has noted that dexmedetomidine, an α‐2 adrenergic receptor stimulant widely used for sedation during CA, causes coronary vasospasms, supports this perspective.^5^ However, it is unclear whether clinical coronary spastic angina (CSA) predisposes patients to develop dexmedetomidine‐induced coronary vasospasms. Here, we describe a patient who experienced dexmedetomidine‐induced coronary vasospasms during CA but had a negative acetylcholine provocation test.

A 68‐year‐old man with drug refractory, symptomatic persistent AF was referred to our hospital for CA. He had a medical history of hypertension and hypercholesteremia and took valsartan 80 mg/day, azelnidipine 8 mg/day, and rosuvastatin calcium 2.5 mg/day. He had never experienced any chest pain that suggested clinical CSA. His body height was 173 cm and body weight was 80 kg, and he had smoked half a pack‐per‐day of cigarettes for several years but quit over 40 years ago. Echocardiography showed a normal left ventricular ejection fraction (73%) and no valvular disease. Enhanced CT scans demonstrated no thromboses in the left atrial appendage or calcifications around the coronary arteries.

The 12‐lead electrogram (ECG) showed normal ST‐segments before the sedation (Figure 1A), and his blood pressure was 113/79 mmHg and heart rate 71 beats per minute. Deep sedation was performed with an injection of pentazocine 30 mg, bolus injection of propofol (1.25 mg/kg), and loading infusion of dexmedetomidine (4 μg/kg/h). Following the initial sedation, a maintenance dose of propofol (0.4–1.0 mL/kg/h) and dexmedetomidine (0.4 μg/kg/h) was continued to maintain the bispectral index (BIS) level at almost 40–60. (Figure 2) Immediately after inserting three wires into the femoral vein, ST segment elevation was noted in the inferior leads (Figure 1B), suggesting acute myocardial ischemia. The blood pressure dropped to 71/49 mmHg and the heart rate decreased to 49 beats per minute. During coronary vasospasm, the BIS level ranged between 28 and 50. An urgent CAG revealed severe stenosis at the midportion of the RCA (Figure 1D). In response to an injection of 0.5 mg of isosorbide dinitrate into the RCA, about 1 min later, the ST elevation in inferior leads and stenosis both became reversed (Figure 1C,E). A continuous injection of nicorandil was started. After recovery of the vital signs to normal, a pulmonary vein isolation, electrical isolation of the posterior left atrial wall and CTI ablation were completed without any ST segment re‐elevation.

**FIGURE 1:**
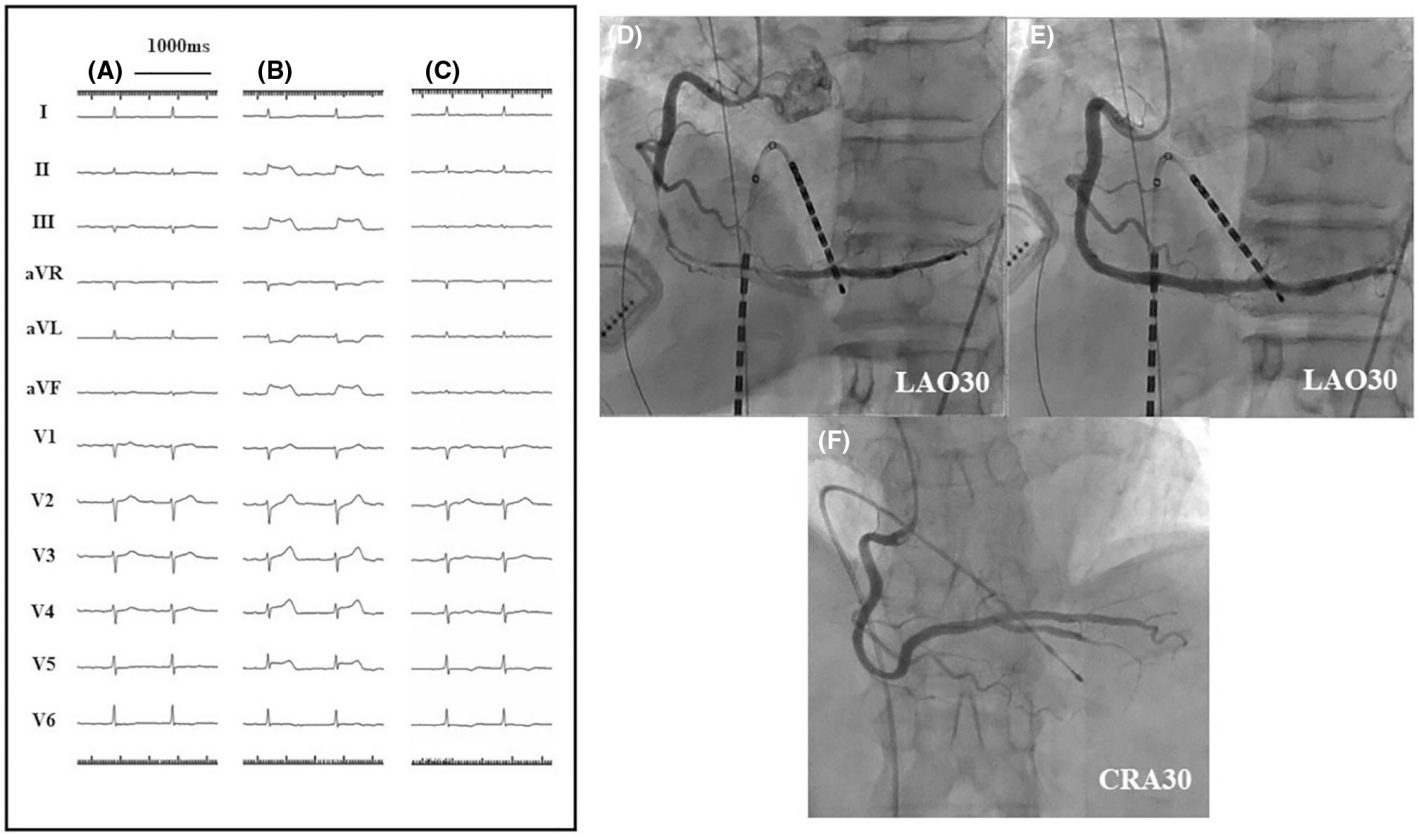
Changes in the electrocardiogram and coronary angiogram during catheter ablation and the results of the acetylcholine provocation test. (A) ECG at baseline. (B) The ECG after the initial loading of dexmedetomidine exhibited ST‐T elevation in the inferior leads with reciprocal changes. (C) The ECG after injecting isosorbide dinitrate into the RCA. (D) Urgent CAG showing severe stenosis in the midportion of the RCA. (E) CAG after injecting isosorbide dinitrate into the RCA. The severe stenosis completely recovered. (F) An acetylcholine provocation test with 50 μg, performed 72 h after the RF application showed negative findings. CRA, cranial; ECG, electrocardiogram; LAO, left anterior oblique.

**FIGURE 2:**
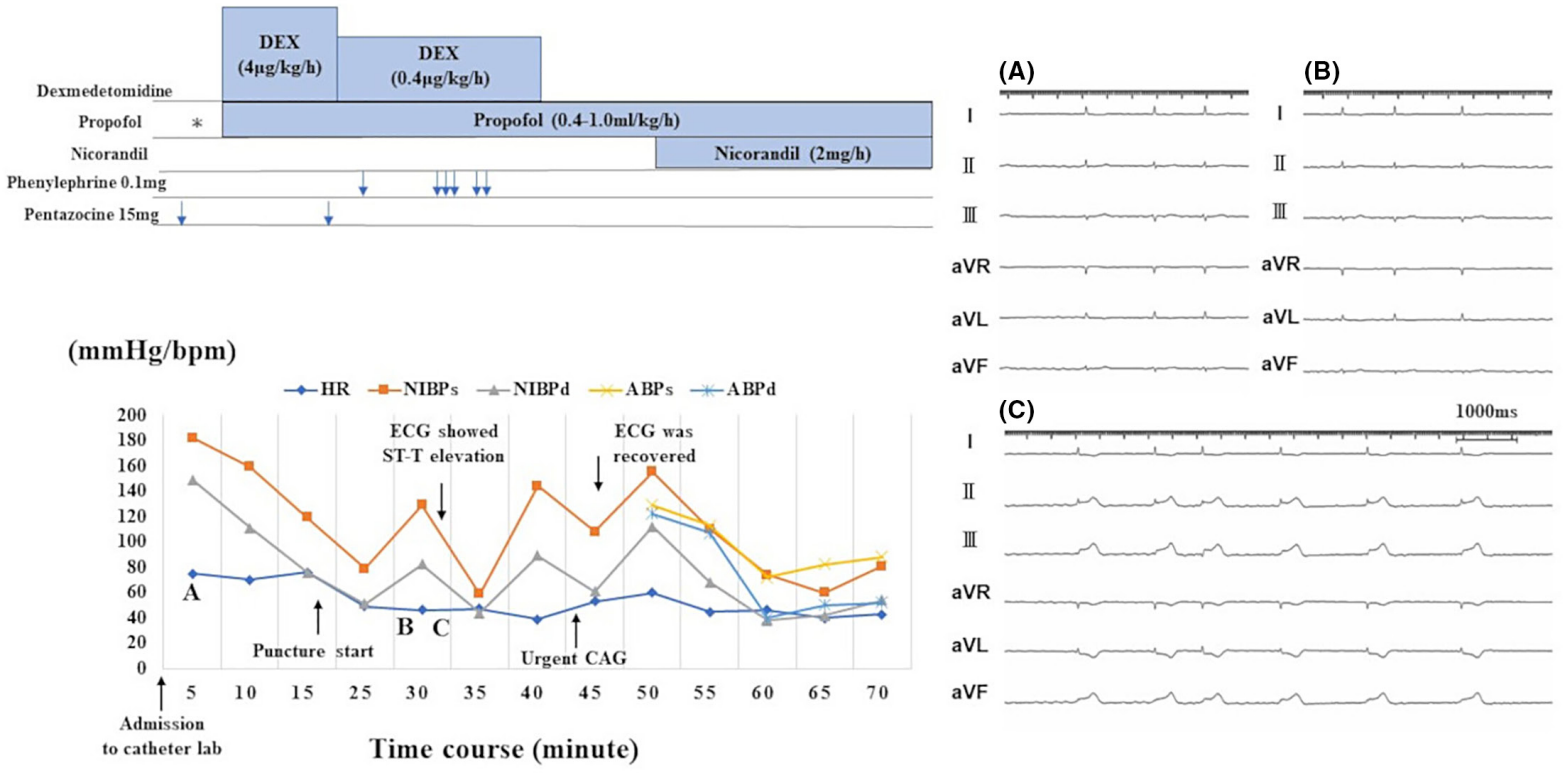
The summary of the procedural course. The summary of the procedural course during the ablation. The blood pressure suddenly dropped after the initial loading of DEX. The coronary vasospasms were diagnosed by an urgent CAG and improved after a coronary injection of isosorbide dinitrate. *: bolus injection of propofol (1.25 mg/kg). Blue arrows: timing and number of drugs administered. (A) The limb lead ECG at admission to catheter lab. (B) The limb lead ECG soon after we administered 0.1 mg of phenylephrine remained normal. (C) A long duration recording of the limb lead ECG during ST‐T elevations. There were no instances of complete atrioventricular block. ABP, invasive atrial blood pressure; bpm, beats per minutes; d, diastolic; DEX, dexmedetomidine; HR, heart rate; NIBP, non‐invasive blood pressure; s, systolic.

One day after the procedure, a 12‐lead ECG no longer showed any ST segment elevation. The continuous injection of nicorandil and oral azelnidipine were stopped. Forty‐eight hours later, an acetylcholine provocation test was performed to diagnose CSA. We administered 50 μg of acetylcholine to both the right and left coronary arteries, but the test results were negative (Figure 1F). Based on the results, we concluded that this patient did not have predisposing CSA. We restarted the azelnidipine and added no other medications for CSA.

Several reports have noted that coronary vasospasms may occur during CA. Because it is commonly caused by direct thermal effects or an autonomic neuronal imbalance, coronary vasospasms before ablation are rare. Frui et al. reported a case with coronary spasms caused by a dexmedetomidine infusion before an ablation.^3^ Because provocation tests were not performed in the previous literature, the relationship between drug‐induced coronary spasms and the existing clinical coronary spasms, which are provoked by acetylcholine, remains unknown. To the best of our knowledge, our case was the first to describe coronary vasospasms induced by a dexmedetomidine infusion in a patient with negative findings of an acetylcholine provocation test. Dexmedetomidine stimulates the α‐2 adrenergic receptors, which mediate the vasoconstriction of the coronary circulation.^5^ The initial loading dose highly stimulated the α‐2 adrenergic receptors and induced coronary spasms and ST‐T elevation. However, our patient never experienced any CSA or any chest pain before the ablation. Furthermore, he took a calcium channel blocker as a hypertensive drug. Thus, a detailed investigation is needed to assess the necessity of long‐term treatment for CSA. The present case emphasized that care should be taken to monitor the vital signs and ischemic changes in the ECG after initiating a dexmedetomidine infusion, even when a patient does not have any past medical history of CSA.

While a previous report about coronary vasospasms during ablation revealed positive acetylcholine provocation test results,^2^ the present case had negative results. The present case indicated that coronary vasospasms during procedures do not necessarily correlate with clinical CSA, suggesting the potential usefulness of a CSA provocation test. Moreover, a CSA provocation test may be useful for those who develop coronary vasospasms during procedures regardless of the cause.

## CONFLICT OF INTEREST STATEMENT

The authors declare no conflict of interest for this article.

## CONSENT FOR PUBLICATION

Patient consent for publication was obtained.
